# Unintended consequences of vaginal misoprostol use in a patient with hydatidiform mole: A case report

**DOI:** 10.1097/MD.0000000000050978

**Published:** 2026-10-09

**Authors:** Hyung Joon Yoon, Yong Jung Song, Dong Soo Suh, Kyung Un Choi, Ki Hyung Kim

**Affiliations:** a Department of Obstetrics and Gynecology, Pusan National University School of Medicine, Busan, Republic of Korea; b Biomedical Research Institute, Pusan National University Hospital, Busan, Republic of Korea; c Research Institute for Convergence of Biomedical Science and Technology, Pusan National University Yangsan Hospital, Yangsan, Republic of Korea; d Department of Pathology, Pusan National University School of Medicine, Busan, Republic of Korea.

**Keywords:** expulsion, hydatidiform mole, hysterectomy, misoprostol

## Abstract

**Rationale:**

Hydatidiform mole (HM) is usually managed by suction curettage, while misoprostol is not recommended as a method of medical evacuation for molar pregnancy because of concerns regarding incomplete evacuation and postmolar gestational trophoblastic neoplasia. The use of misoprostol for cervical ripening before hysterectomy in patients with known molar pregnancy is also not supported by established evidence. This case highlights the potential risks of unintended misoprostol use in gynecologic procedures involving molar tissue.

**Patient concerns:**

A 49-year-old woman presented with vaginal bleeding and imaging findings suspicious for HM. Her serum β-human chorionic gonadotropin (β-hCG) level was markedly elevated at 589,830 mIU/mL.

**Diagnoses:**

Histopathological examination of the expelled molar tissue confirmed a partial HM. Although serum β-hCG initially declined markedly following complete expulsion, it subsequently increased at 9 to 10 weeks, indicating persistent or recurrent trophoblastic disease.

**Interventions:**

Hysterectomy was initially planned, and 400 μg of vaginal misoprostol was administered preoperatively for cervical ripening to facilitate uterine manipulator insertion. Unexpectedly, the patient experienced uterine contractions and complete expulsion of the molar tissue the following morning. Because of the subsequent rise in serum β-hCG, hysterectomy was performed. Persistent low-level β-hCG after surgery prompted methotrexate (MTX) treatment.

**Outcomes:**

Following MTX treatment, serum β-hCG levels plateaued, and the patient was considered to have MTX-resistant disease. Multi-agent chemotherapy with EMA-CO (etoposide, methotrexate, actinomycin D, cyclophosphamide, and vincristine) was subsequently administered.

**Lessons:**

This case illustrates the potential unintended consequences of using misoprostol in a patient with known molar pregnancy. Although complete expulsion of the molar tissue occurred, the subsequent development of postmolar gestational trophoblastic neoplasia and the need for multi-agent chemotherapy indicate that this should not be considered a favorable therapeutic outcome. Misoprostol should not be used for cervical ripening when hysterectomy is planned as the primary treatment for molar pregnancy. This case highlights the importance of avoiding inappropriate off-label use of misoprostol in the presence of molar tissue and emphasizes the need for careful post-evacuation β-hCG surveillance regardless of the initial clinical outcome.

## 1. Introduction

The incidence of hydatidiform mole (HM) ranges from 0.57 to 2 cases per 1000 pregnancies.^[1]^ Extremes of maternal age are a well-established risk factor for complete HM, likely related to abnormal gametogenesis and fertilization of oocytes at these reproductive age limits.^[2]^ Patients most commonly present with vaginal bleeding during the second trimester.

Suction evacuation and curettage, preferably under ultrasound guidance, remain the standard treatment for molar pregnancy when fertility preservation is desired. For women who have completed childbearing, hysterectomy is a suitable alternative, providing definitive sterilization and reducing the risk of persistent disease by eliminating potential myometrial invasion, thereby decreasing the likelihood of chemotherapy.^[3]^ In contrast, medical induction of labor with agents such as misoprostol is not recommended, as it is associated with greater maternal morbidity and an increased risk of postmolar gestational trophoblastic neoplasia (GTN).^[4,5]^ Nonetheless, conflicting evidence exists; Aminimoghaddam et al reported that vaginal misoprostol administered prior to suction curettage for cervical ripening in 50 patients with molar pregnancy was not associated with major complications.^[6]^

We describe a rare case of HM that was unintentionally and completely expelled following vaginal misoprostol administration. Although misoprostol is not recommended by current guidelines, this case highlights the possibility of complete molar evacuation after its incidental use.

## 2. Case report

A 49-year-old woman (gravida 3, para 1) presented with vaginal bleeding and imaging findings suspicious for HM. Her last menstrual period was 12 + 3 weeks earlier. Her past history included a cesarean section and thyroid cancer surgery without adjuvant therapy. She was stable on examination (blood pressure 115/68 mm Hg, heart rate 101 bpm), with mild anemia (hemoglobin 10.5 g/dL). Ultrasonography and computed tomography revealed an enlarged uterus (15.4 × 8.8 cm) with a heterogeneous, honeycomb-like mass (8.9 × 6.8 cm; Fig. 1 and B). Serum β-human chorionic gonadotropin (β-hCG) was markedly elevated (589,830 mIU/mL). Thyroid function showed elevated T3 (262.6 ng/dL), normal FT4, and suppressed thyroid-stimulating hormone. No metastases were detected. Hysterectomy was planned. Vaginal misoprostol (400 μg) was administered preoperatively for cervical ripening, given her history of cesarean delivery and absence of prior vaginal delivery. At 4:00 am, she experienced abdominal pain and expelled a large amount of uterine tissue (Fig. 2). Follow-up ultrasonography confirmed complete evacuation with no residual intrauterine mass (Fig. 3). Subsequently, vaginal bleeding subsided, and no chest discomfort was noted. She was discharged in the afternoon of the same day with outpatient follow-up. Histopathology confirmed partial mole with enlarged hydropic villi and trophoblastic proliferation. P57 immunostaining was positive. Serum β-hCG declined to 6556 mIU/mL 2 weeks after expulsion but rose again at 9 and 10 weeks (49.10 and 113.00 mIU/mL, respectively). The patient opted for hysterectomy, taking into account a prior surgical attempt. Postoperatively, β-hCG levels at 1, 2, and 3 weeks were 41.70, 40.40, and 35.70 mIU/mL, respectively, prompting methotrexate (MTX) administration intramuscularly weekly. However, β-hCG plateaued (32.10, 31.80, 42.00, and 38.00 mIU/mL at 1 to 4 weeks post-MTX), necessitating EMA-CO chemotherapy (etoposide, methotrexate, actinomycin D, cyclophosphamide, and vincristine). Thereafter, β-hCG levels returned to normal. At 11 months of postoperative follow-up, the patient remained disease-free. This study was approved by the Institutional Review Board of Pusan National University Hospital (IRB #2511-005-156). Written informed consent was obtained from the patient for publication of this case report and any accompanying images.

**Figure 1.**
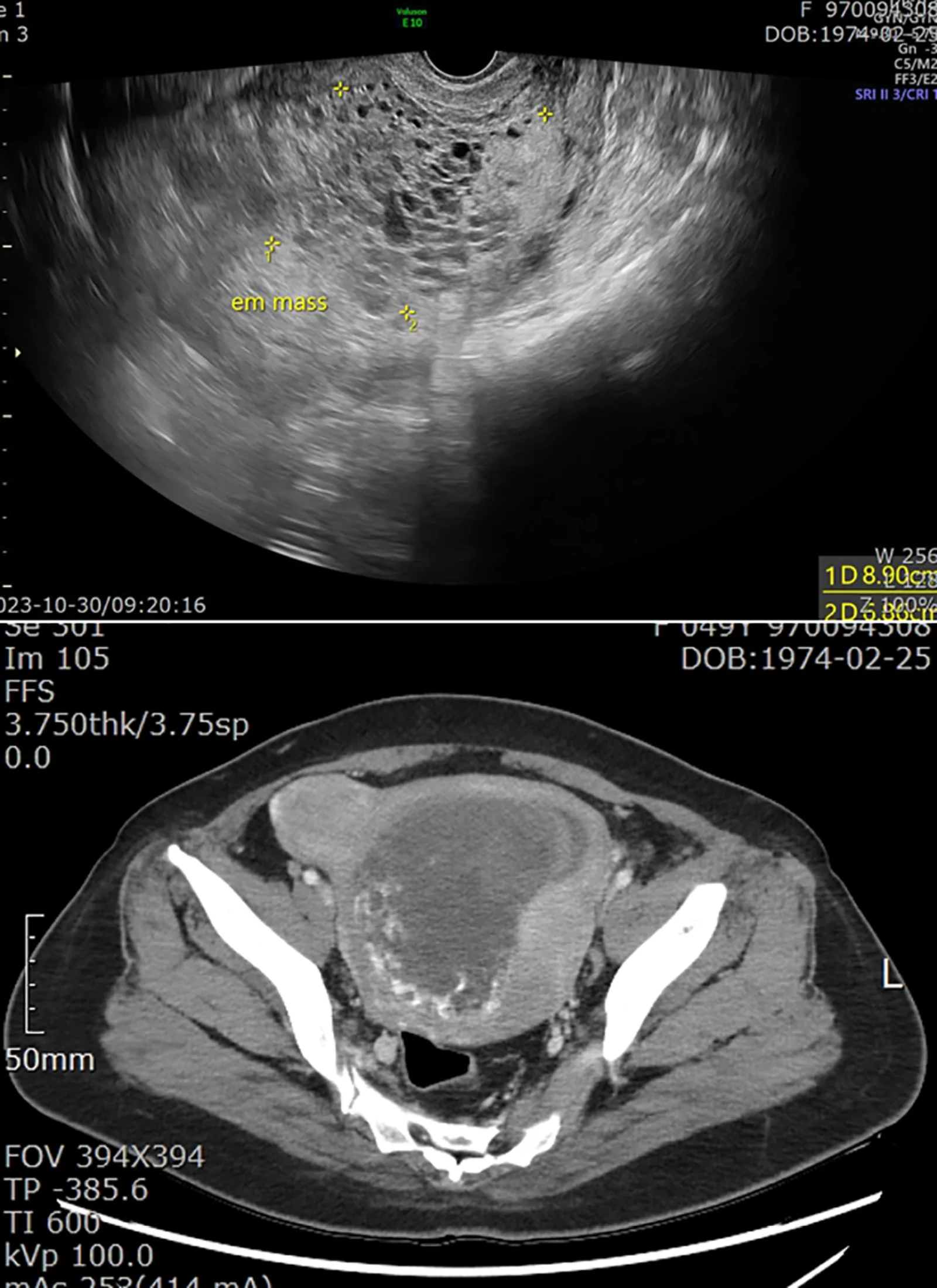
Radiological findings. Transvaginal ultrasonography and contrast-enhanced CT revealed an enlarged uterus containing a heterogeneously enhancing, expansile mass with a honeycomb appearance that distended the endometrial cavity. CT = computed tomography.

**Figure 2.**
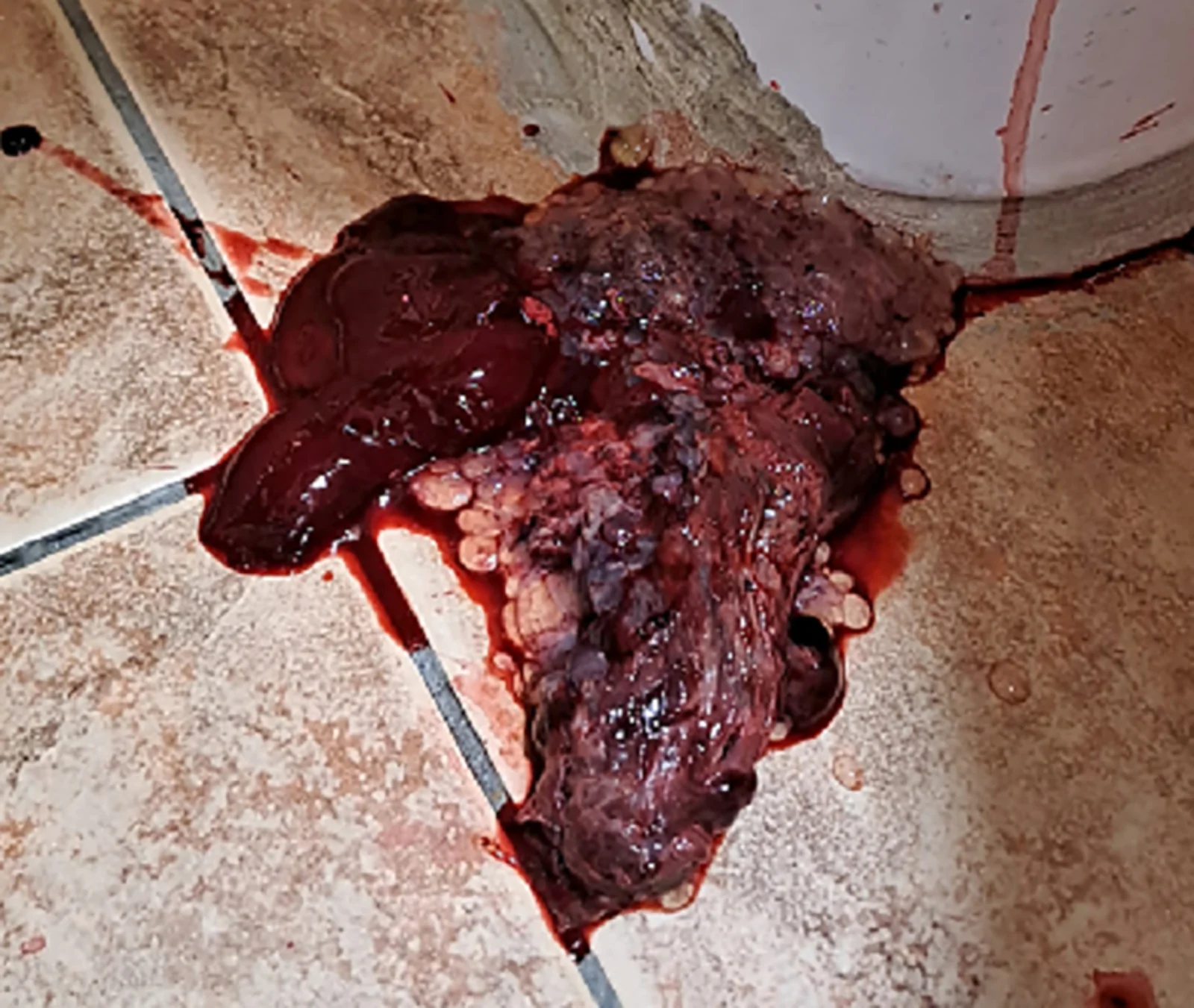
Expelled molar tissue following vaginal misoprostol administration.

**Figure 3.**
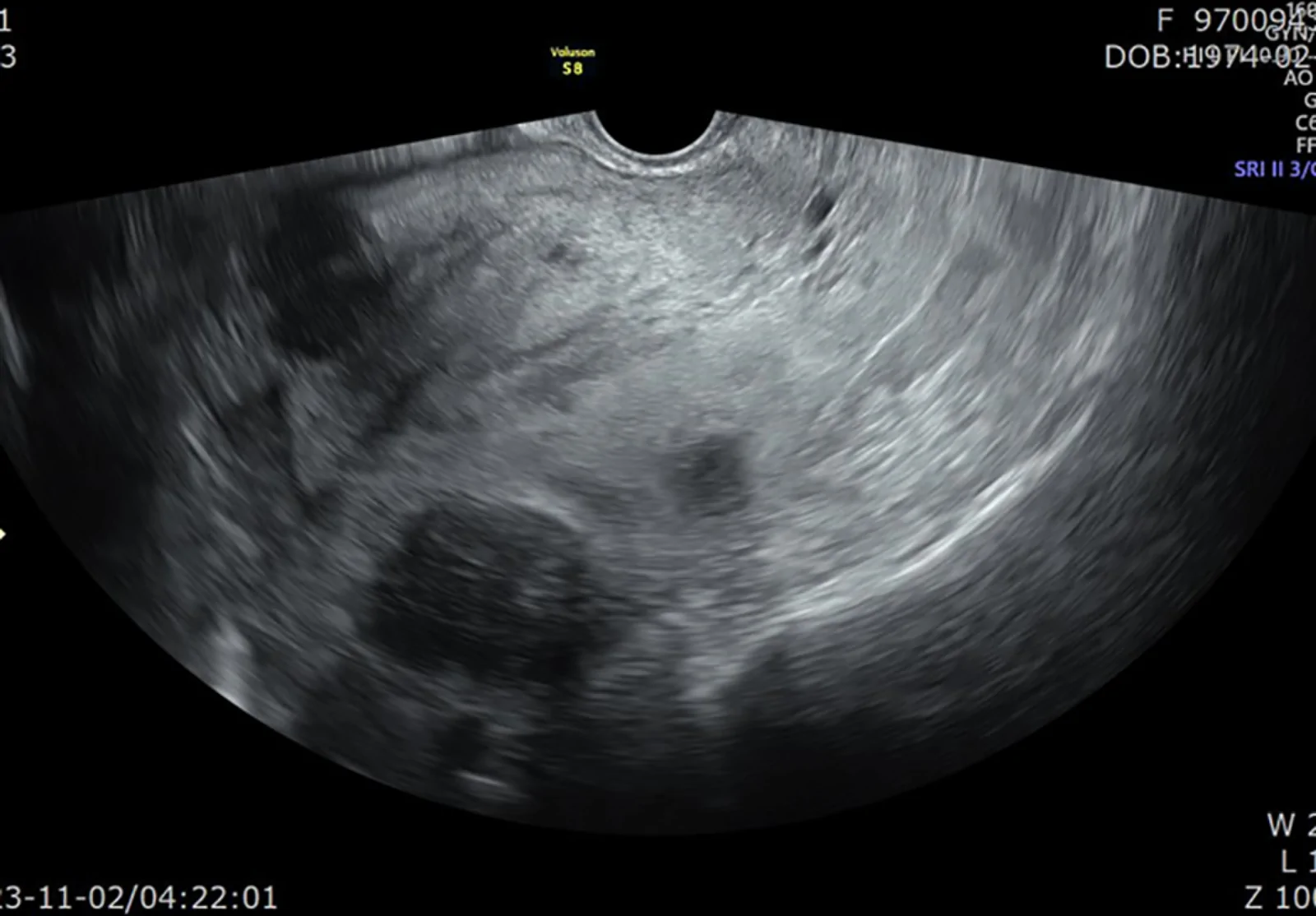
Transvaginal ultrasonography revealed no residual mass within the uterine cavity.

## 3. Discussion

This case highlights the potential unintended consequences of misoprostol use in gynecologic procedures involving molar tissue. Misoprostol is a prostaglandin E1 analogue with both cervical ripening and uterotonic effects. Although it is widely used for cervical preparation and uterine evacuation in various obstetric and gynecologic settings, its use in molar pregnancy is not recommended as a method of medical evacuation because of concerns regarding incomplete evacuation and postmolar GTN.^[4,5]^ In the present case, misoprostol was not intended to induce evacuation; rather, it was administered for cervical ripening before planned hysterectomy. Nevertheless, its uterotonic effect was followed unexpectedly by complete expulsion of the molar tissue.

Standard HM treatment is suction evacuation or hysterectomy with β-hCG surveillance.^[7]^ Suction curettage is preferred in women desiring fertility, whereas hysterectomy is often advised in patients ≥40 years without fertility wishes. Evidence on whether hysterectomy significantly lowers GTN incidence remains mixed.^[5,8]^ Thus, the decision to perform primary hysterectomy in an older patient should be individualized after consideration of the patient’s age, reproductive wishes, clinical condition, and potential risks and benefits. In the present case, hysterectomy was initially planned in a 49-year-old woman who had completed childbearing.

Misoprostol is widely used in obstetrics and gynecology for its cervical ripening and uterotonic properties, indicated in medical abortion, miscarriage management, labor induction, intrauterine fetal death, and postpartum hemorrhage.^[9]^ However, routine use in molar pregnancy is discouraged due to risks of incomplete evacuation and GTN,^[4]^ and it is not clearly addressed in gynecologic and obstetric guidelines. Although some studies have reported that vaginal misoprostol administered before suction curettage did not result in major complications in selected patients with molar pregnancy,^[6]^ these findings do not establish the safety of misoprostol for cervical preparation before hysterectomy in patients with known molar pregnancy. In addition, previous reports have described cases in which molar tissue was expelled following misoprostol administration.^[10–12]^ Therefore, the potential uterotonic effects of misoprostol should be carefully considered whenever molar tissue is present.

Conversely, some studies have reported safer outcomes. Aminimoghaddam et al used vaginal misoprostol before suction curettage in 50 molar pregnancies without major complications, supporting its safety for cervical ripening.^[6]^ Two additional cases were managed with vaginal misoprostol followed by ultrasound-guided suction evacuation: 1 complete mole with a live fetus and 1 partial mole with live fetuses. An initial 800 µg dose, followed by 400 µg every 4 hours (up to 6 doses), led to expulsion after 3 to 4 doses, with suction curettage confirming complete evacuation.^[11]^ Another report described oral misoprostol in a triplet pregnancy with partial HM and 2 live fetuses; 400 µg every 4 hours (3 doses) enabled cervical dilation and expulsion of 2 fetuses, 2 placentas, and the molar tissue.^[12]^

Misoprostol can be used for cervical preparation prior to uterine instrumentation, such as intrauterine device insertion. Numerous studies have also evaluated its use before hysteroscopy across diverse populations, including nulliparous and postmenopausal women, as well as those with a history of cesarean delivery. A recent systematic review confirmed that misoprostol facilitates cervical dilation and reduces procedure time.^[13]^ Consequently, misoprostol is routinely used in clinical practice to improve outcomes of hysteroscopic procedures by preparing the cervix. In premenopausal women without prior vaginal delivery, a systematic review and meta-analysis showed that misoprostol significantly increases cervical width and decreases the need for additional mechanical dilation.^[14]^ However, these studies concern hysteroscopic procedures and do not provide evidence supporting the use of misoprostol for cervical ripening to facilitate insertion of a uterine manipulator during hysterectomy.

In our institution, vaginal misoprostol had been used in selected patients without a history of vaginal delivery to facilitate uterine manipulator insertion during laparoscopic hysterectomy when a narrow cervical canal was considered likely to cause technical difficulty. Cervical dilation is additionally performed using a Hegar dilator after anesthesia. In the present case, 400 µg of vaginal misoprostol was administered preoperatively on the evening before surgery to aid cervical ripening. Unexpectedly, at 4:00 am the following day, the patient experienced lower abdominal pain and expelled a large amount of uterine tissue (Fig. 2). Subsequent ultrasonography confirmed complete evacuation with no residual intrauterine mass (Fig. 3). In retrospect, the use of misoprostol for cervical ripening to facilitate uterine manipulator insertion during hysterectomy was not appropriate in this patient with known molar pregnancy. The medication was administered with the intention of achieving cervical ripening rather than inducing uterine evacuation, and insufficient consideration was given to its potential uterotonic effect in the presence of molar tissue. This experience led us to reconsider this practice and to avoid the use of misoprostol for cervical ripening in patients with molar pregnancy when hysterectomy is planned.

Postmolar GTN is typically detected through serial β-hCG surveillance, often without clinical symptoms. Monitoring β-hCG every 1 to 2 weeks is essential for early detection and timely management. Surgical intervention may be required in some cases. Minimally invasive hysterectomy provides oncologic outcomes comparable to open abdominal hysterectomy in GTD, with added benefits of reduced blood loss and shorter hospitalization. A plateau or rise in β-hCG after initial decline indicates GTN. The risk of postmolar GTN rises with maternal age, reported at 37% in women over 40 and up to 56% in those over 50, compared with 15% to 20% in younger patients.^[5]^

In the present case, the patient was a 49-year-old premenopausal woman with a relatively high risk of developing GTN. Complete expulsion of molar tissue was achieved following the administration of vaginal misoprostol, with a subsequent marked decline in β-hCG levels. However, during weeks 9 to 10, β-hCG levels rose again after a prior decrease. Low-risk GTN is usually treated with single-agent chemotherapy, while hysterectomy may be considered in perimenopausal women without fertility desire, potentially reducing chemotherapy cycles and toxicity.^[15]^ In the present case, a hysterectomy was subsequently performed, but β-hCG levels plateaued within 1 to 3 weeks postoperatively. The patient was started on MTX but demonstrated resistance to the drug. Ultimately, multi-agent chemotherapy with EMA-CO was initiated. Importantly, the present case does not establish a causal relationship between misoprostol administration and the subsequent development of GTN or resistance to MTX.

In summary, although complete expulsion of the molar tissue occurred without immediate major complications, the subsequent clinical course cannot be regarded as a favorable therapeutic outcome. The present case does not support the therapeutic use of misoprostol for molar pregnancy and should not be interpreted as evidence that misoprostol can serve as an alternative method of molar evacuation. Rather, this case highlights 2 potential risks: the risks and limitations of primary hysterectomy for molar pregnancy in older patients and the risks associated with the off-label use of misoprostol in the presence of molar tissue. In particular, misoprostol should not be used for cervical ripening to facilitate uterine manipulator insertion when hysterectomy is planned as the primary treatment for molar pregnancy. We acknowledge that its use in this setting represented an inappropriate and erroneous application of the treatment policy. This case therefore serves as a cautionary example of the potential unintended consequences of off-label misoprostol use and underscores the importance of adhering to evidence-based management strategies and maintaining close post-evacuation β-hCG surveillance.

## Author contributions

**Conceptualization:** Hyung Joon Yoon.

**Formal analysis:** Yong Jung Song.

**Investigation:** Yong Jung Song.

**Methodology:** Dong Soo Suh.

**Project administration:** Kyung Un Choi.

**Supervision:** Dong Soo Suh, Kyung Un Choi, Ki Hyung Kim.

**Validation:** Dong Soo Suh, Ki Hyung Kim.

**Writing – original draft:** Hyung Joon Yoon.

**Writing – review & editing:** Ki Hyung Kim.
